# The ‘PSILAUT’ protocol: an experimental medicine study of autistic differences in the function of brain serotonin targets of psilocybin

**DOI:** 10.1186/s12888-024-05768-2

**Published:** 2024-04-25

**Authors:** Tobias P. Whelan, Eileen Daly, Nicolaas A. Puts, Paula Smith, Carrie Allison, Simon Baron-Cohen, Ekaterina Malievskaia, Declan G. M. Murphy, Grainne M. McAlonan

**Affiliations:** 1 Department of Forensic and Neurodevelopmental Sciences, Institute of Psychiatry, Psychology & Neuroscience, King’s College London, London, UK; 2 COMPASS Pathfinder Ltd, London, UK; 3 Medical Research Council Centre for Neurodevelopmental Disorders, King’s College London, London, UK; 4 Autism Research Centre, Department of Psychiatry, University of Cambridge, Cambridge, UK; 5 NIHR-Maudsley Biomedical Research Centre at South London and Maudsley NHS Foundation Trust and the Institute of Psychiatry, Psychology and Neuroscience, King’s College London, London, UK

**Keywords:** Autism, Psilocybin, Psychedelics, Serotonin, Pharmacology, Neuroimaging

## Abstract

**Background:**

The underlying neurobiology of the complex autism phenotype remains obscure, although accumulating evidence implicates the serotonin system and especially the 5HT_2A_ receptor. However, previous research has largely relied upon association or correlation studies to link differences in serotonin targets to autism. To directly establish that serotonergic signalling is involved in a candidate brain function our approach is to change it and observe a *shift* in that function.

We will use psilocybin as a pharmacological probe of the serotonin system in vivo. We will directly test the hypothesis that serotonergic targets of psilocybin – principally, but not exclusively, 5HT_2A_ receptor pathways—function differently in autistic and non-autistic adults.

**Methods:**

The ‘PSILAUT’ “shiftability” study is a case–control study autistic and non-autistic adults. How neural responses ‘shift’ in response to low doses (2 mg and 5 mg) of psilocybin compared to placebo will be examined using multimodal techniques including functional MRI and EEG. Each participant will attend on up to three separate visits with drug or placebo administration in a double-blind and randomized order.

**Results:**

This study will provide the first direct evidence that the serotonin targets of psilocybin function differently in the autistic and non-autistic brain. We will also examine individual differences in serotonin system function.

**Conclusions:**

This work will inform our understanding of the neurobiology of autism as well as decisions about future clinical trials of psilocybin and/or related compounds including stratification approaches.

**Trial registration:**

NCT05651126.

## Background

Autism spectrum disorder (hereafter referred to as ‘autism’) is a neurodevelopmental condition characterised by differences in social interaction and communication, repetitive or restricted patterns of behaviour, and sensory differences [1]. Although the neurobiological underpinnings of the diverse autistic phenotype remain obscure, accumulating evidence strongly supports involvement of the serotonin system. Identifying differences in the serotonin system in autistic individuals may reveal novel mechanisms to be targeted pharmacologically to benefit those who seek support.

### The serotonin system in autism

There is increasing evidence from association and/or correlation studies linking serotonin to autism. First, polymorphisms in genes for serotonin synthesis, transporters and receptors are associated with autism [2, 3]. Second, elevated whole blood serotonin levels are also reported in one-third of autistic individuals [4, 5]. Third, our team has previously reported that acutely elevating serotonin levels with a single dose of selective-serotonin reuptake inhibitor (SSRI) citalopram differentially affects autistic brain function. For example, citalopram produces sustained activation of brain regions associated with facial expression processing in autistic adults, but not in controls [6].

More specifically, there is also evidence implicating specific serotonin receptors in autism, especially the 5HT_2A_ receptor. This receptor is involved in dendritic maturation, neuronal differentiation and the regulation of brain-derived neurotrophic factor levels during development [7]. At the circuit level, 5HT_2A_ receptor signalling is thought to enhance neural plasticity [8] and increases cortical glutamate and thalamic GABA levels [9]. The receptor is expressed throughout the cortex but especially in regions related to sensorimotor integration [10] and the so-called default mode network responsible for “self” and “other” processing [11]. Thus, through these key processes that shape neuronal architecture and neurotransmission, serotonin influences lower-order systems (e.g. sensory) through to higher-order processes (e.g. whole-brain connectivity) as they emerge. As a result, early perturbations in the serotonin system, such as alterations in 5HT_2A_ receptor-signalling, may influence subsequent brain developmental outcomes. Indeed the HT2RA gene is a candidate gene associated with autism [12–15]. However, although lower cortical 5HT_2A_ receptor binding has been reported to correlate with social communication differences in autism [16], to date there have been no studies that have directly tested whether 5HT_2A_ receptor pathways function differently in autistic and non-autistic people.

### Measuring ‘Shift’ in the serotonin system in autism

To do this, we have developed a ‘“shiftability” paradigm [17] to examine how ‘foundational’ mechanisms of brain function such as neural responses to sensory stimuli or whole-brain network connectivity captured using electroencephalography (EEG) and functional magnetic resonance imaging (fMRI) are modulated by targeted pharmacological probes. These measures are biologically informative and may be used across a wide range of ages and, in many cases, also back-translate to animal models to inform future target identification and engagement studies [18–23].

**Fig. 1:**
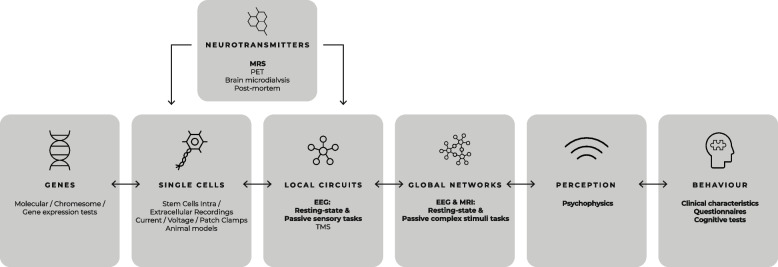
The organisational levels of information processing in the brain and methodologies that probe each level. Methodologies used to detect ‘shift’ that are included in our ‘PSILAUT’ protocol and discussed here are shown in bold. Bidirectional arrows represent interaction between organisation levels (adapted from Ahmad & Ellis, 2022 [24])

The range of measures used to capture ‘shift’ and their target level of brain organisation is shown in Fig. 1.

### Targeting 5HT_2A_ receptor in autism using psilocybin

The only way to directly test if a neurosignalling system functions differently in one group of individuals compared to another is to experimentally manipulate it (for example using a pharmacological probe) and observe a ‘shift’ in function. In humans unlike preclinical model systems, it is of critical importance that the choice of pharmacological probe is safe and has minimal side effects. However, few (if any) neuropsychiatric drugs used in people are entirely specific in their biological effects. With this caveat in mind, in this protocol we selected psilocybin (4-phosphoryloxy-*N.N*-dimethyltryptamine), as a pharmacological probe of the 5HT_2A_ receptor in autistic and non-autistic adults.

Psilocybin is a classic psychedelic compound produced by several species of mushrooms, including so-called “magic mushrooms”. Psilocybin is rapidly metabolised into its active component psilocin [25]. Psilocin is a 5HT_2A_ receptor agonist but also binds several serotonin receptors, including 5HT_7_, 5HT_2B_, 5HT_1D_, 5HT_6_, 5HT_5_, 5HT_2C_ & 5HT_1B_ receptors in decreasing order of reported affinity [26]. Prior studies have used relatively high doses of psilocybin to explore the effects of psychedelics on the brain. However, in the planned study we will use lower doses (2 mg and 5 mg) in our “shiftability” protocol to assess brain responses so as not to cause a marked psychedelic experience. This dose range is expected to generate a ‘shift’ in brain function based on evidence using positron emission topography that similar doses of psilocybin engage 5HT_2A_ receptors [27]; and low dose psilocybin has been shown to be sufficient to alter cognition and obsessive–compulsive behaviour [28, 29]. Low doses of the serotonergic psychedelic lysergic acid diethylamide (LSD) and psilocybin-containing mushrooms also acutely alter brain resting-state fMRI and EEG indices in the non-autistic population [30, 31], as well as neural responses to sensory stimuli [32]. Therefore, we expect that 2 mg and 5 mg of psilocybin will expose functional differences in the serotonin system targeted by psilocybin in autistic and non-autistic individuals using the experimental medicine approach outlined in this protocol.

### Overall design of the ‘PSILAUT’ study

The study is an Investigator-Initiated Study sponsored by King’s College London and co-Sponsored by South London and Maudsley NHS Foundation Trust (SLaM). It is part funded by COMPASS Pathfinder Ltd with infrastructure support from the NIHR-Maudsley Biomedical Research Centre at South London and Maudsley NHS Foundation Trust and King’s College London. COMPASS Pathfinder Ltd are donating psilocybin (as “COMP360”). The study is a case–control study with a pseudo-randomised, double-blind, placebo-controlled, cross-over design. We have capacity to recruit up to 70 adult participants to accommodate participant drop-out and/or data quality screening with the goal of *n* = 30 autistic adults (half female) and *n* = 30 non-autistic adults (half female), who will be matched by age and sex. All participants will provide written informed consent. Participants will be asked to attend 3 visits in total and on each visit they will receive either placebo or one of two single doses (2 mg or 5 mg) of oral synthetic COMP360 psilocybin. Participants and the researchers accompanying the visit will be blind to allocation. The order of administration of placebo and psilocybin will also be pseudo-randomised by the Chief Investigator using a randomisation tool to help generate a list (e.g. Random.org, Randomness and Integrity Services Ltd.) then modified manually to ensure a reasonable balance of individuals in each cell are allocated each of the 3 possible administration orders throughout the duration study (for example, to avoid a ‘run’ of placebo first visits, should different groups/sexes be harder to recruit and result in different cell sizes). Thus, there are 3 possible orders of administration allocated:

1. Visit 1, placebo; Visit 2, 2 mg psilocybin; Visit 3, 5 mg psilocybin
2. Visit 1, 2 mg psilocybin; Visit 2, 5 mg psilocybin; Visit 3, placebo
3. Visit 1, 2 mg psilocybin; Visit 2, placebo; Visit 3 5 mg psilocybin

The lowest dose of psilocybin will always precede the higher dose. This allows us to unblind in the case of unwanted side effects (such as a significant increase in blood pressure) or unwanted experience and potentially omit the higher dose visit. We also explain that there will be more chance of side effects with the higher dose in our informed consent process and if a participant only wishes to attend for the placebo and lower dose visit, we can exclude the higher dose from the randomisation (i.e. they would attend a total of 2 visits only). Thus, a ‘drop-out’ would be considered a participant that did not attend all 3 visits and has missing data for at least one condition.

### Ethical considerations

This study will take place at the Institute of Psychiatry, Psychology and Neuroscience (IoPPN) at De Crespigny Park, SE5 8AF, London, United Kingdom. Our study does not address safety or clinical efficacy and the UK Medicines and Health Regulatory Authority (MHRA) has confirmed that our protocol is therefore not a clinical trial of an Investigational Medicinal Product (IMP) as defined by the EU Directive 2001/20/EC.

The authors assert that all procedures contributing to this work comply with the ethical standards of the relevant national and institutional committees on human experimentation and with the Helsinki Declaration of 1975, as revised in 2013. All procedures involving human participants were approved by Dulwich Research Ethics Committee (Reference: 21/LO/0795) and the study protocol was peer reviewed during the ethical review process. As clinicaltrials.gov accepts a range of study designs, because definitions of study types using pharmacological probes differ in different jurisdictions, and in the interest of transparency, we have preregistered the study (Identifier: NCT05651126). We emphasize that in this study, to ensure individuals can provide full informed consent, we will be recruiting autistic and non-autistic adults without intellectual disability and the doses of psilocybin used will be low to limit likelihood of marked psychedelic experiences.

### Community engagement

Community engagement was led by the Autism Research Centre at Cambridge University in 2019, prior to designing the study. Three hundred and thirty-one autistic adults were asked about their attitudes to psilocybin research in autism. It was made clear that the research team were not aiming to ‘treat’ autism itself and that the research would not proceed if there was not clear support for it from the autism community. The majority of respondents supported exploratory studies using psilocybin.

Of the 331 autistic adult respondents, 41% were ‘very interested’ and 28% were ‘somewhat interested’. For example: *“I am very interested in helping with pioneering new approaches… and feel it is high time proper research was done in this area”.* There was also caution however as 25% were ‘not interested’ or ‘not at all interested’. Therefore, rather than proceed immediately to a conventional clinical trial, this study was designed to provide further information on the brain functions targeted by low dose psilocybin and understand any differences between autistic and non-autistic adults. Our hope is that this study will establish a firmer neurobiological evidence base to inform potential opportunities for the development of psilocybin as a pharmacological support option.

**Fig. 2:**
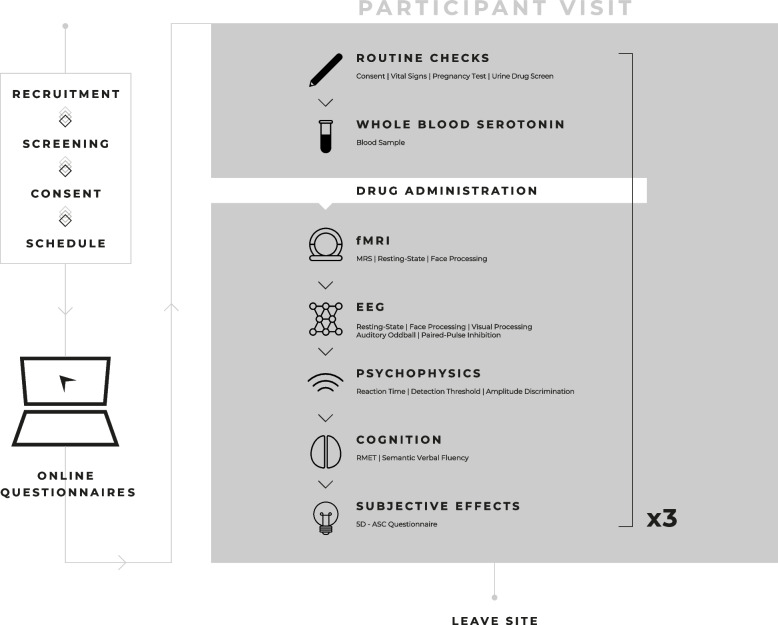
'PSILAUT' recruitment and study procedures. Autistic and non-autistic participants will be recruited from existing local research databases, advertising on the King’s College London website and wider dissemination of study information. Participants are welcome to self-refer. Autistic participants will also be recruited from clinical contacts from South London and Maudsley NHS trust, local and national support groups and via the Cambridge Autism Research Database (CARD), managed by our collaborators at the Autism Research Centre, University of Cambridge. Interested participants will be sent an information sheet and screened via video call or phone for eligibility according to the inclusion and exclusion criteria prior to the first visit. Written consent will be sought after inclusion criteria are confirmed and the participant is then assigned to a study schedule. Participants will be provided with login details to an online platform (Delosis Ltd., London) to complete a battery of questionnaires remotely. Participants will visit the study site on three separate occasions. A blood sample will be collected on one of the three visits for quantification of whole blood serotonin levels. Each participant will complete an MRI scan session to acquire a structural, resting-state functional MRI scan and a face emotion processing task. The EEG paradigm will include resting-state and functional activation during a face processing, auditory oddball and visual processing. Psychophysical tasks will be collected prior to a cognitive battery which will include the ‘reading the mind in the eyes’ (RMET), probabilistic reversal learning (PRT), both of which will be delivered using PsyTools (Delosis Ltd., London) and a semantic verbal fluency task. The 5-dimensional altered states of consciousness (5D-ASC) questionnaire will then be completed to quantify any subjective effects experienced by participants

Stakeholder engagement also included a press release by King’s College London, South London and Maudsley NHS Foundation Trust and COMPASS Pathways (https://ir.compasspathways.com/news-releases/news-release-details/compass-pathways-fund-study-comp360-psilocybin-autistic-adults), as well as articles and interviews across several platforms including The Economist (https://www.economist.com/psychedelics-pod), Psychology Today [33], Technology Networks [34] and The BBC (https://www.bbc.co.uk/programmes/m001j45x), which received positive feedback. Study findings will be continue to be widely disseminated in forums, publications and presentations involving stakeholders in the autistic community, study participants, researchers, industry and clinicians (Fig. 2).

## ‘PSILAUT’ protocol measures

Each study visit will last approximately 4–5 h in total. Although given the number of measures, the data collected is time permitting and cognitive tasks at the end of the protocol may be omitted, for example. We will aim to collect spectroscopy and resting-state MRI at 60min and 70min post-dose at T_max_ [27], respectively; EEG at ~ 2 h post-dose & psychophysics at ~ 3 h post-dose. We have established the tolerability and feasibility of this study design in autistic adults as we have conducted similar studies previously with several different pharmacological probes [6, 21, 35–38]. Indeed, many of our participants have also attended our studies in the past and are well-informed about the visit procedures.

### Inclusion & exclusion criteria

A comprehensive baseline characterisation will be obtained. An expert clinical diagnosis of autism from a recognised UK assessment service will be accepted. This may be supported by the Autism Diagnostic Interview-Revised [39] where an appropriate informant is available. An Autism Diagnostic Observation Schedule [40] will be used to support diagnosis, but if it has already been used to inform the diagnostic assessment in adulthood, it will not be repeated. Participants with ASD of a known genetic cause (e.g. Fragile X syndrome) were excluded. Other inclusion criteria include being over 18 years old; the ability to provide informed consent; no cooccurring psychiatric illness such as major mood disorder or psychotic illness; no history of seizures or diagnosis of epilepsy and no physical illness such as high blood pressure. Participants taking medications known to affect serotonin (such as selective serotonin-reuptake inhibitors) will be excluded. Those taking stimulants will be eligible and asked to ideally exclude on the day of testing or else ensure they take it the same way on each visit. These and any other potential medication use will be included as covariates in analyses and/or statistical analyses rerun excluding data from individuals taking medication to establish the extent to which medication use drives any potential findings.

### Baseline characterisation

Additional baseline questionnaires will quantify autistic traits (e.g. social behaviour or sensory differences), relevant cognitive domains (e.g. intolerance of uncertainty and behavioural flexibility) and the symptomology of co-occurring psychiatric conditions.

### Neurometabolites

#### Magnetic Resonance Spectroscopy (MRS)

An MRS Hadamard Encoding and Reconstruction of MEGA-Edited Spectroscopy (HERMES) sequence [41] will be collected during the MRI scan for the dorsal medial prefrontal cortex region. HERMES permits the quantification of levels of metabolites in the living brain and is focused on estimating GABA and Glutamate-glutamine markers of E/I balance. For the purposes of our study, given the evidence that E-I pathways are modulated by 5HT_2A_ receptor action in animal models [9], we will be able to examine the impact of psilocybin on these tissue level measures of E-I balance.

### Local circuits

#### EEG

##### Resting-state

High-density (64-channel) EEG data will be collected during the resting-state, metrics from which local circuit activity can be derived such as beta and gamma band power/frequency [24]. Oscillations in the beta frequency band at rest, for example, are associated with inhibitory neurotransmitter levels in sensorimotor cortex [42]. This, and other EEG-derived metrics such as aperiodic activity, are considered a proxy measure for E/I balance in vivo [24]. Hence, we will be able to examine the impact of psilocybin on these dynamic measures of E/I balance.

#### Passive sensory tasks

##### Visual domain

A visual processing task (contrast saturation) in which steady-state evoked potentials (SSVEPs) are elicited by passive surround suppression stimuli will be conducted. We have shown that SSVEPs during this task are altered in autism [21]. 5HT_2A_ receptors are particularly highly expressed in the primary visual cortex [11], and their agonism alters visual response amplitudes and surround suppression in mouse primary visual cortex [43]. In humans, we expect visual processing to be altered by 5HT_2A_ receptor activation given that the marked visual perceptual changes robustly induced with higher doses of psychedelics are blocked by pretreatment with the 5HT_2_ receptor antagonist, ketanserin [44].

##### Auditory domain

A conventional auditory oddball paradigm of mismatch negativity (MMN) [45] will be used to passively measure ‘repetition suppression’ (or habituation) to repetitive auditory stimuli and response to an unexpected ‘deviant’ stimulus (the event-related mismatch negativity MMN response). We and others have observed less repetition suppression in both eight-month-old infants who go on to receive a diagnosis or autism, and adults with a diagnosis of autism [19, 46]. Thus, this signal appears linked to autism across infancy to maturity. The impact of autism on the event-related MMN is less consistent and varies with age [47–49]. The latter may in part be due to differences in the serotonin system, as the MMN response can be modulated by acute elevation of serotonin levels by the highly selective SSRI escitalopram [50]. In this study we will test the prediction that psilocybin alters both sensory suppression and MMN in autism differently compared to controls.

### Global networks

#### Resting-state

##### EEG

Oscillatory power will be assessed across multiple frequency bands during the resting-state. This will include electrodes over key brain regions implicated in autism such as those belonging to the default mode network (DMN) [51]. Reduced oscillatory power over DMN regions using electrophysiological approaches following 5HT_2A_ receptor activation by psilocybin has been reported previously [52]. Functional connectivity analyses (e.g. within and between brain networks) can also be derived from EEG, and this will complement connectivity analyses from resting-state fMRI.

##### MRI

Participants will undergo a structural and functional MRI scan. Scans will be acquired on a 3.0 Tesla MR Scanner (General Electric Premier). A fMRI scan with a multiband 4 sequence will be acquired during the resting-state, multiband 4 is preferable for connectivity analyses [53]. In addition, multiband sequences will considerably reduce the repetition time (TR), therefore they have the advantage of allowing dynamic functional connectivity analyses. Autistic differences in both ‘averaged’ functional connectivity and dynamic functional connectivity have been reliably reported across different datasets [20, 54]. Functional connectivity of brain networks in neurotypical individuals has also be shown to be acutely modulated by 5HT_2A_ receptor activation [55]. Notably, psilocybin alters dynamic functional connectivity, mediated by 5HT_2A_ receptor agonism [56]. It facilitates state transitions and more temporally diverse brain activity in neurotypical individuals[57]. Our study will be the first to examine the effects of psilocybin on conventional and dynamic functional metrics in autistic individuals.

#### Task-dependent MRI and EEG

##### MRI

fMRI studies of face emotion processing in autism have produced inconsistent results. In the largest study to date, no differences between autistic and non-autistic individuals in fMRI response to facial expressions of emotion were observed [58]. However, we have recently examined the fMRI response to facial expressions of emotion in a social brain network before and after administration of the SSRI, citalopram. We reported that the dynamics of the response to faces is different in autism, in that serotonin reuptake inhibition slows habituation [6]. Consistent with this, blockade of 5HT_2A_ receptors causes an ‘opposite’ effect and reduces neural responses to emotional faces during fMRI in neurotypical individuals [59]. Therefore, we expect that psilocybin will alter the dynamics of face emotion processing, but differently in autistic individuals.

##### EEG

Event-related potentials (ERPs) in response to face stimuli will also be assessed during EEG. The N170 component, a neural response present at 170ms following the presentation of facial stimuli and can be modulated by 5HT_2A_ receptor activation [44, 60, 61]. An altered N170 response is associated with social communication differences in autism and may have utility as a stratification marker that is amenable to support [62]. It is also going to be the first prognostic biomarker for autism (or any neurodevelopmental or psychiatric condition) to be approved by regulatory agencies [62]. The incorporation of this task in our protocol therefore will be an important test of whether an autism biomarker can be modified pharmacologically.

### Perception

Psychophysical approaches are structured approaches in which stimulus characteristics are tightly controlled, and they provide robust, objective measures of sensory sensitivity by estimating perceptual metrics [63, 64]. Serotonin has been directly implicated in tactile perception, such as in affective touch, by studies using tryptophan depletion (which acutely reduces central serotonin levels) alongside psychophysical approaches [65]. Tactile detection threshold and amplitude discrimination will be assessed, as differences in tactile thresholds have already been reported in autism and are associated with outcomes [66]. As processing of tactile stimuli is also known to be perturbed by 5HT_2A_ receptor agonism with psilocybin [67], we also expect to elicited functional differences following psilocybin in autistic and non-autistic individuals in this paradigm.

### Additional measures

#### Questionnaires

The 5-dimensional altered states of consciousness (5D-ASC) questionnaire [68] will be completed on each visit following the completion of study procedures to quantify the subjective effects of psilocybin, which are primarily mediated by the 5HT_2A_ receptor [69].

#### Cognitive battery

‘Theory of mind’ (i.e. cognitive empathy, the ability to understand and take into account the mental state of another individual) can be investigated using the ‘reading the mind in the eyes’ (RMET) task [70]. Other cognitive processes in which differences are observed in autism such as language and executive and reward-related functioning (e.g. flexible choice behaviour) will be assessed with a verbal fluency task and probabilistic reversal learning task, respectively [71, 72].

#### Peripheral biochemistry

Participants will be asked to provide a blood sample on one visit prior to placebo/drug administration (their preference). Whole blood serotonin levels will be determined for each individual, given that elevated levels are present in one-third of individuals with autism [4, 5]. This will allow us to explore whether any ‘shift’ in brain function in response to psilocybin depends on overall serotonin ‘tone’ as indexed by proxy.

### Data analyses

The overarching goal of our analyses is to assess whether we see a ‘shift’ by psilocybin in autistic and non-autistic individuals for each modality. Both parametric and nonparametric statistical analyses will be used to test hypotheses that the serotonergic targets of psilocybin functioning differently in autistic individuals. Given the heterogeneity of the autistic population and our prior observations that there is a wide range of pharmacological responses in both autistic and non-autistic individuals, we will calculate individual ‘shift’ for each modality, and what characteristics (e.g. clinical scores, questionnaire responses, whole blood serotonin) these are associated with. Although we will generate and analyse data from single modalities, post-hoc we will also explore multimodal metrics (i.e. associations between modalities) to understand how ‘shifts’ detected across multiple organisational levels are inter-related.

### Power analyses

We will use a within-subject, repeated-measures design with a placebo condition so that each subject is their own control, thus increasing statistical power. Results from our prior neuroimaging studies using pharmacological challenge were successful in detecting group differences in MRI metrics with sample sizes of *n* =  < 20 [6, 35–38, 73–75]. This implies an effects size (expressed as Cohen’s d) in excess of 1.2. In sensory tasks a sample size of *n* = 16 per cell is estimated to achieve 80% power to detect a medium effect (0.5) at a = 0.05; this has been achieved even in mixed sex groups of 20 participants or fewer. This reflects the literature in which significant group differences are evident even in mixed sex groups of 20 participants or fewer (e.g. *n* = 16) [76–78]. Nevertheless, we aim for *n* = 30 per group (approximately half female in both groups). Our design relies upon participants attending for repeat test sessions. Thus, there is a chance that participants may ‘drop-out’ and need to be replaced, this is accommodated with our ethically approved total sample size of *n* = 70.

### Limitations

Our protocol requires active participation despite the passive nature of several of our tasks. For example, participants will wear an EEG cap and will be asked to remain focused on the screen during the presentation of sensory stimuli. This may limit generalisability across ages or to autistic individuals with higher support needs.

Even in the proposed adult cohort, participants may get restless or become distracted. Hence, we have included concurrent eye tracking during EEG to control for the potential confound of participant variability in fixation on the screen. However, many of our tasks require minimal or no response from participants and so they are less likely to be impacted by confounds such as individual cognitive difficulties. We hope that this way, should our indices prove worthy of further investigation and/or incorporation in (for example) clinical trials, they will be more accessible for individuals who may otherwise be excluded from drug development studies.

## Discussion

Our “shiftability” paradigm aims to determine how different organisational scales of brain function are modulated by the serotonin system, in particular the 5HT_2A_ receptor pathway when activated by psilocybin. It will test the hypothesis that the serotonin system targeted by psilocybin is different in autistic and non-autistic people. The results will expand our understanding of brain biology in autistic and non-autistic individuals.

Our work will inform a more personalized medicine approach to autism. Not everyone in our study will respond the same way to psilocybin. Whilst our study design may reveal differences in the response to psilocybin at the group-level, crucially, our prior studies have shown that its constituents are also sensitive to the potential variability at the individual-level. For example, we have been able to plot individual ‘shifts’ in GABA and glutamate spectroscopy, task-dependent and resting-state fMRI measures in response to several drug challenges [6, 36–38, 74], and calculated an individual ‘sensitivity index’ across the different sensory modalities examined in response to a GABA challenge [21, 46]. Thus, by investigating individual biology in both autistic and non-autistic people, this experimental medicine approach may help identify autistic individuals whose serotonin system functions no differently from non-autistic people, and who might therefore not be expected to show a clinical response in a clinical trial. And vice versa; those who respond biologically to psilocybin challenge might ultimately benefit clinically. To date, all clinical trials for the core features of autism have failed to reach their primary endpoint. This is in large part because they have included participants based on diagnosis alone and measured outcomes, often without evaluating mechanisms in the same cohort. Existing pharmacological options available for autistic people that target the serotonin system are mainly selective serotonin reuptake inhibitors (reviewed in Howes et al., 2017 [79]). These show limited efficacy in core domains. For example, there is some evidence for their utility in addressing repetitive behaviours in adults, as assessed using obsessive compulsive symptom outcome measures [80]. They have also been used to manage co-occurring mental health conditions such as anxiety and depression. Unfortunately, these medications can be poorly tolerated [81], highlighting the need for novel options, and the importance of identifying serotonergic mechanisms to target in the autistic brain specifically prior to clinical trials. Therefore, in depth pharmacological ‘profiling’ adopting some of the methods described here may help avoid the unnecessary expense and likely failure of clinical trials and facilitate the discovery of novel pharmacological support options for those who would like that choice.

## Data Availability

No datasets were generated or analysed during the current study.

## Abbreviations

5D-ASC: 5-Dimensional altered states of consciousness
5HT: 5-Hydroxytryptamine
DMN: Default mode network
EEG: Electroencephalography
E/I: Excitation/Inhibition
ERP: Event-related potential
GABA: Gamma-aminobutyric acid
HERMES: Hadamard Encoding and Reconstruction of MEGA-Edited Spectroscopy
EEG: Electroencephalography
MRI: Magnetic resonance imaging
MRS: Magnetic Resonance Spectroscopy
RMET: Reading the mind in the eyes test
SSRI: Selective-serotonin reuptake inhibitor
SSVEP: Steady-state evoked potential
